# Surface modification of pig endothelial cells with a branched heparin conjugate improves their compatibility with human blood

**DOI:** 10.1038/s41598-017-04898-w

**Published:** 2017-06-30

**Authors:** Anjan K. Bongoni, Evelyn Salvaris, Sofia Nordling, Nikolai Klymiuk, Eckhard Wolf, David L. Ayares, Robert Rieben, Peetra U. Magnusson, Peter J. Cowan

**Affiliations:** 1 Immunology Research Centre, St. Vincent’s Hospital Melbourne, Victoria, Australia; 2 Department of Immunology, Genetics and Pathology, Uppsala University, Uppsala, Sweden; 3 Institute of Molecular Animal Breeding and Biotechnology, Ludwig-Maximilian University, Munich, Germany; 4 Revivicor Inc., Blacksburg, VA USA; 5 Department of Clinical Research, University of Bern, Bern, Switzerland; 6 Department of Medicine, University of Melbourne, Victoria, Australia

## Abstract

Corline Heparin Conjugate (CHC), a compound of multiple unfractionated heparin chains, coats cells with a glycocalyx-like layer and may inhibit (xeno)transplant-associated activation of the plasma cascade systems. Here, we investigated the use of CHC to protect WT and genetically modified (GTKO.hCD46.hTBM) pig aortic endothelial cells (PAEC) in two pig-to-human *in vitro* xenotransplantation settings. Model 1: incubation of untreated or hTNFα-treated PAEC with 10% human plasma induced complement C3b/c and C5b-9 deposition, cellular activation and coagulation activation in WT and GTKO.hCD46.hTBM PAEC. Coating of untreated or hTNFα-treated PAEC with CHC (100 µg/ml) protected against human plasma-induced endothelial activation and damage. Model 2: PAEC were grown on microcarrier beads, coated with CHC, and incubated with non-anticoagulated whole human blood. Genetically modified PAEC significantly prolonged clotting time of human blood (115.0 ± 16.1 min, p < 0.001) compared to WT PAEC (34.0 ± 8.2 min). Surface CHC significantly improved the human blood compatibility of PAEC, as shown by increased clotting time (WT: 84.3 ± 11.3 min, p < 0.001; GTKO.hCD46.hTBM: 146.2 ± 20.4 min, p < 0.05) and reduced platelet adhesion, complement activation, coagulation activation and inhibition of fibrinolysis. The combination of CHC coating and genetic modification provided the greatest compatibility with human blood, suggesting that pre-transplant perfusion of genetically modified porcine organs with CHC may benefit post-transplant xenograft function.

## Introduction

In organ transplantation, the vascular endothelium of the donor organ is the first point of contact with the recipient’s blood. Vascular endothelial cells normally express an anti-inflammatory and anti-coagulant surface, crucial for the natural anticoagulation of blood. These properties are maintained by several mechanisms including the expression of membrane-bound regulatory molecules and the presence of the glycocalyx, a multicomponent layer of proteoglycans and glycoproteins covering the luminal surface of the endothelium. Both endothelium- and plasma-derived soluble molecules integrate into this network layer. Heparan sulfate (HS, 50–90%) and chondroitin sulfate (10–20%) are the most abundant oligosaccharides in the glycocalyx^1^. Due to the heterogeneity of its composition and its location, the glycocalyx plays a critical role as a protective barrier in maintaining vessel wall permeability and modulating blood cell-vessel wall interactions^2–5^. The transplant process activates and injures graft endothelium, causing shedding of the glycocalyx and loss of regulators, creating a pro-inflammatory, pro-coagulant environment^6^. While the graft eventually recovers, this early insult can contribute to primary non-function and cause long-term detrimental effects.

Pig-to-human xenotransplantation has the potential to solve the chronic and increasing shortage of human donor organs. However, the vascular endothelium of a pig xenograft is more sensitive to human blood than that of a human allograft for a number of reasons, including the presence of anti-pig antibodies and molecular incompatibilities affecting the regulation of inflammation and coagulation^7, 8^. Genetic modification of the donor pig to remove antibody targets (α1,3-galactosyltransferase gene knockout [GTKO]), regulate complement activation (expression of human regulatory proteins, CD46, CD55 or CD59), and correct molecular incompatibilities (expression of human thrombomodulin [hTBM]) has improved the survival of porcine xenografts in preclinical models^9^, but does not fully address the problem of endothelial activation and shedding of the glycocalyx. Heparin, a naturally occurring sulfated polysaccharide in the body, has potent anti-coagulant and anti-inflammatory properties and is routinely used to prevent harmful blood clotting. It produces its major anticoagulant effect by accelerating ~2000-fold the inactivation of thrombin and activated factor X by antithrombin^10^. The main limitation of heparin is that in addition to binding to antithrombin, it has a propensity to bind to positively charged plasma proteins and proteins released from platelets and endothelial cells, resulting in a variable anticoagulant response and the phenomenon of heparin resistance^11^.

Corline Heparin Conjugate (CHC) is a macromolecular conjugate composed of multiple (>20) unfractionated heparin chains. Surface-immobilized CHC significantly improves the blood compatibility of biomaterial surfaces by inhibiting coagulation and inflammation^12–15^. Importantly, this unique molecule also binds directly to the surface of living human endothelial cells under physiological conditions, without compromising their biological function^16^. However, CHC binding to pig endothelial cells and its effects on the interaction of the cells with human blood have not been examined.

In this project, we hypothesized that CHC can be used to coat pig aortic endothelial cells (PAEC) with a layer that will protect them from the pro-coagulant and pro-inflammatory environment induced by xenotransplantation. We tested this hypothesis by investigating whether coating of wild-type (WT) and GTKO.hCD46.hTBM PAEC with CHC inhibited coagulation and inflammation mediated by *in vitro* incubation with human plasma (model 1) or non-anticoagulated whole human blood (model 2).

## Results

### Human plasma induces shedding of the glycocalyx by PAEC

**Figure 1:**
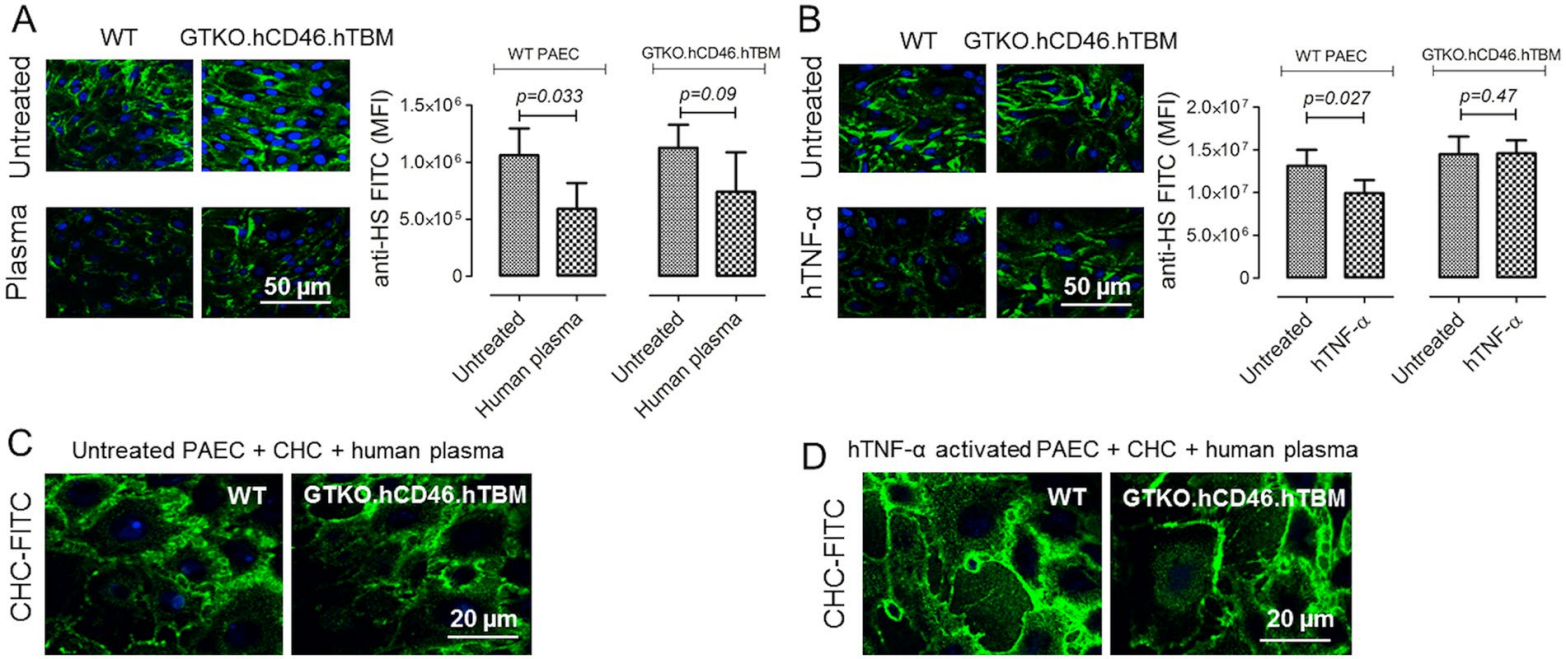
(**A,B**) Heparan sulfate (HS) expression on untreated and human plasma-treated PAEC. WT and GTKO.hCD46.hTBM PAEC were treated either with (**A**) 10% human plasma for 4 hrs or (**B**) 100 ng/ml hTNFα for 2 hrs and stained for HS expression (green) and nuclei (blue) and visualized by confocal microscopy; mean fluorescence intensity (MFI) of anti-HS FITC was quantitated using Image J software. Incubation of WT PAEC with human plasma or hTNFα induced a significant loss of HS expression (both p < 0.05). GTKO.hCD46.hTBM PAEC incubated with human plasma for 4 hrs displayed some loss of HS (not statistically significant), whereas treatment with hTNFα showed no change in HS expression. Statistical significance was measured by unpaired t-test; data are mean ± SD of three independent experiments. Scale bar: 50 µm. (**C,D**) Persistence of FITC-CHC binding to PAEC after 4 hrs treatment with human plasma. (**C**) Untreated or (**D**) hTNFα-treated (100 ng/ml, 2 hrs) WT and GTKO.hCD46.hTBM PAEC were coated with FITC-labeled CHC at 37 °C for 30 min, treated with 10% human plasma for 4 hrs, and analyzed for CHC binding by confocal microscopy. Scale bar: 20 µm.

Untreated WT PAEC strongly expressed HS, and expression was significantly reduced by treatment with 10% human plasma for 4 hrs (p = 0.03 vs. untreated) (Fig. 1A), indicating shedding of the glycocalyx. HS expression on GTKO.hCD46.hTBM PAEC was similar to that on WT PAEC and was also reduced by human plasma treatment, although this did not reach statistical significance (p = 0.09 vs. untreated) (Fig. 1A).

In a separate experiment, WT and GTKO.hCD46.hTBM PAEC were treated with hTNFα (100 ng/ml) for 2 hrs. This treatment induced a significant loss of HS expression in WT PAEC, whereas GTKO.hCD46.hTBM PAEC showed no change (Fig. 1B), which is largely attributed to the fact that hTBM’s lectin-like domain mediates protection against hTNFα^17^. Together, these results demonstrate that hTNFα at high concentration can trigger alterations of the endothelial glycocalyx in WT PAEC.

### Surface coating of PAEC with CHC reduces human plasma-mediated complement deposition and cellular activation

To investigate CHC binding, WT and GTKO.hCD46.hTBM PAEC, starved with RPMI medium plus 0.5% fetal bovine serum (FBS) for 30 min or treated with hTNFα (100 µg/ml) for 2 hrs at 37 °C prior to CHC coating, were incubated with FITC-labeled CHC for 30 min, washed, and examined by confocal microscopy. CHC binding (at 100 µg/ml) was detectable over the whole cell surface, most notably at cell borders, and persisted after 4 hrs incubation with 10% human plasma (Fig. 1C,D).

**Figure 2:**
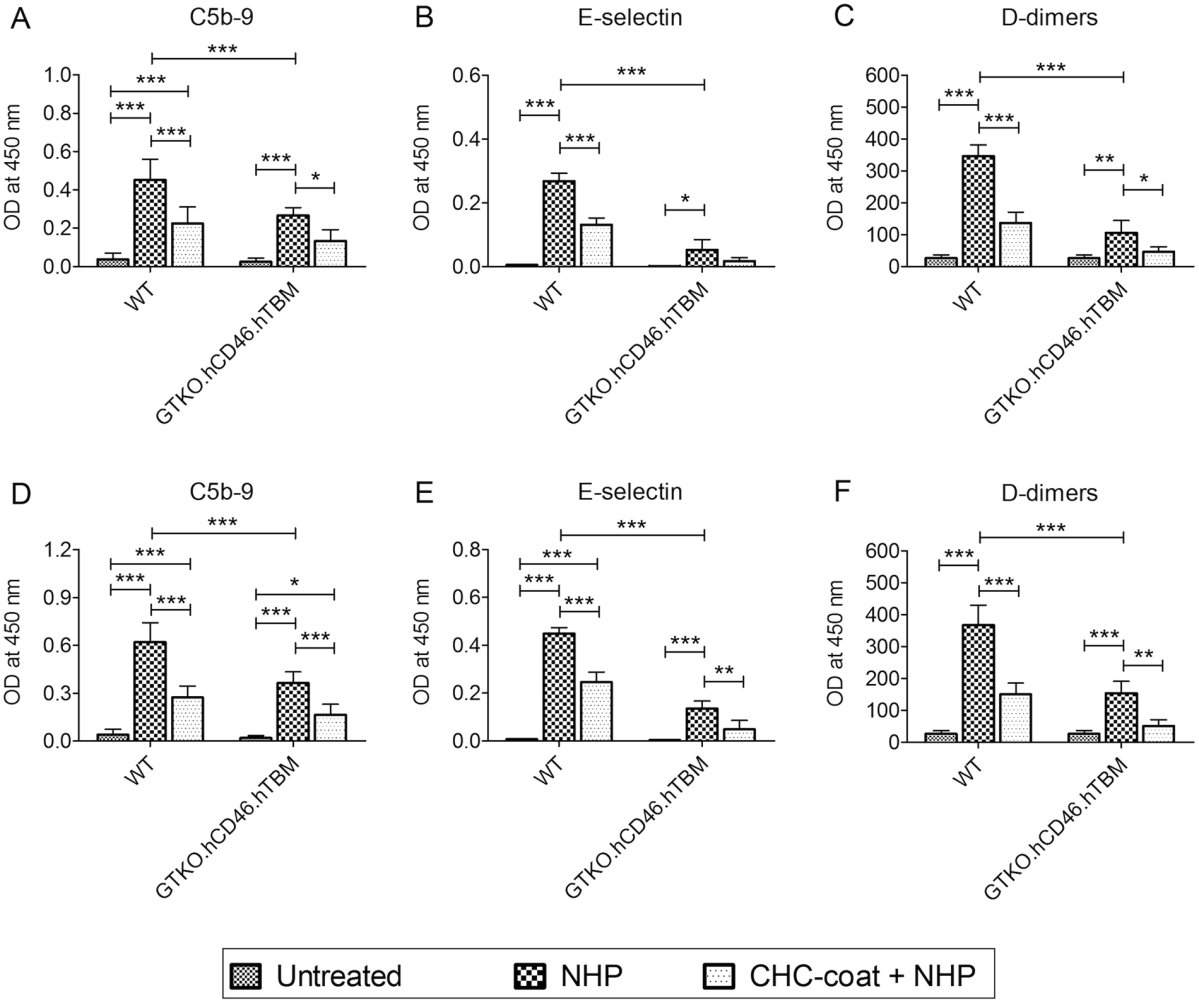
(**A–C**) CHC coating of PAEC reduces complement deposition, cellular activation and coagulation induced by human plasma. Uncoated and CHC-coated PAEC were treated for 4 hrs with 10% human plasma, and assessed by ELISA for (**A**) the deposition of activated complement C5b-9 and (**B**) the expression of the adhesion molecule E-selectin (**B**). (**C**) Supernatants from this assay were measured for D-dimers concentration. CHC coating significantly protected WT PAEC. Uncoated GTKO.hCD46.hTBM PAEC were significantly protected compared to uncoated WT PAEC; coating with CHC further reduced complement deposition and cellular activation, although this was not statistically significant. (**D,F**) CHC coating of hTNFα-activated PAEC reduces complement deposition, cellular activation and coagulation induced by human plasma. WT and GTKO.hCD46.hTBM PAEC were treated with hTNFα (100 ng/ml) for 2 hrs to induce cellular activation and shedding of the endothelial glycocalyx. After, PAEC were coated with CHC and incubated for 4 hrs with 10% human plasma, and assessed for (**D**) complement C5b-9 deposition and (**E**) endothelial E-selectin expression as well as (**F**) D-dimer levels in the supernatants. CHC coating significantly protected hTNFα-activated WT and GTKO.hCD46.hTBM PAEC compared to uncoated PAEC. Significance was measured by one-way ANOVA with Bonferroni correction (*p < 0.05, **p < 0.01, ***p < 0.001). Data are mean ± SD of three independent experiments.

We next tested whether coating with CHC would protect PAEC from the injurious effects of human plasma. PAEC were incubated with 10% human plasma for 4 hrs and assessed for deposition of activated complement (C5b-9) and for complement-mediated endothelial cell activation (upregulation of E-selectin expression). Treatment of ‘CHC-uncoated’ WT and GTKO.hCD46.hTBM PAEC with human plasma significantly increased deposition of complement C5b-9 (Fig. 2A). Human plasma treatment also induced upregulated E-selectin expression in WT and GTKO.hCD46.hTBM PAEC, although the increase was only significant in the WT cells (Fig. 2B). Supernatant samples from the assay showed significantly elevated levels of D-dimers with WT and GTKO.hCD46.hTBM PAEC (Fig. 2C). Complement deposition, cellular activation and soluble coagulation activation markers were lower with GTKO.hCD46.hTBM PAEC than with WT PAEC, indicative of reduced antibody binding and increased regulation of complement and coagulation, consistent with previous observations^17^.

Coating of WT PAEC with CHC significantly reduced human plasma-mediated deposition of C5b-9 (Fig. 2A), E-selectin upregulation (Fig. 2B) and supernatant D-dimers concentration (Fig. 2C). Reductions in these markers were also observed when GTKO.hCD46.hTBM PAEC were coated with CHC, although the decrease was only significant for D-dimers.

We then tested whether coating with CHC would protect hTNFα pre-activated PAEC against the injurious effects induced by hTNFα and human plasma. Confluent WT and GTKO.hCD46.hTBM PAEC were treated with hTNFα (100 ng/ml) for 2 hrs and coated with CHC followed by incubation with 10% human plasma for 4 hrs. Treatment of WT and GTKO.hCD46.hTBM PAEC with hTNFα induced increased C5b-9 deposition (Fig. 2D), E-selectin upregulation (Fig. 2E) and D-dimer levels (Fig. 2F). Coating of hTNFα treated WT PAEC with CHC significantly reduced human plasma-mediated C5b-9 deposition, E-selectin upregulation and D-dimers (Fig. 2D–F). Reductions in these markers were also observed when GTKO.hCD46.hTBM PAEC were coated with CHC. Collectively, these results indicate that CHC can protect PAEC from human plasma-mediated injury by suppressing complement and coagulation activation on the cell surface.

### Surface coating of PAEC with CHC prolongs clotting of human blood

**Figure 3:**
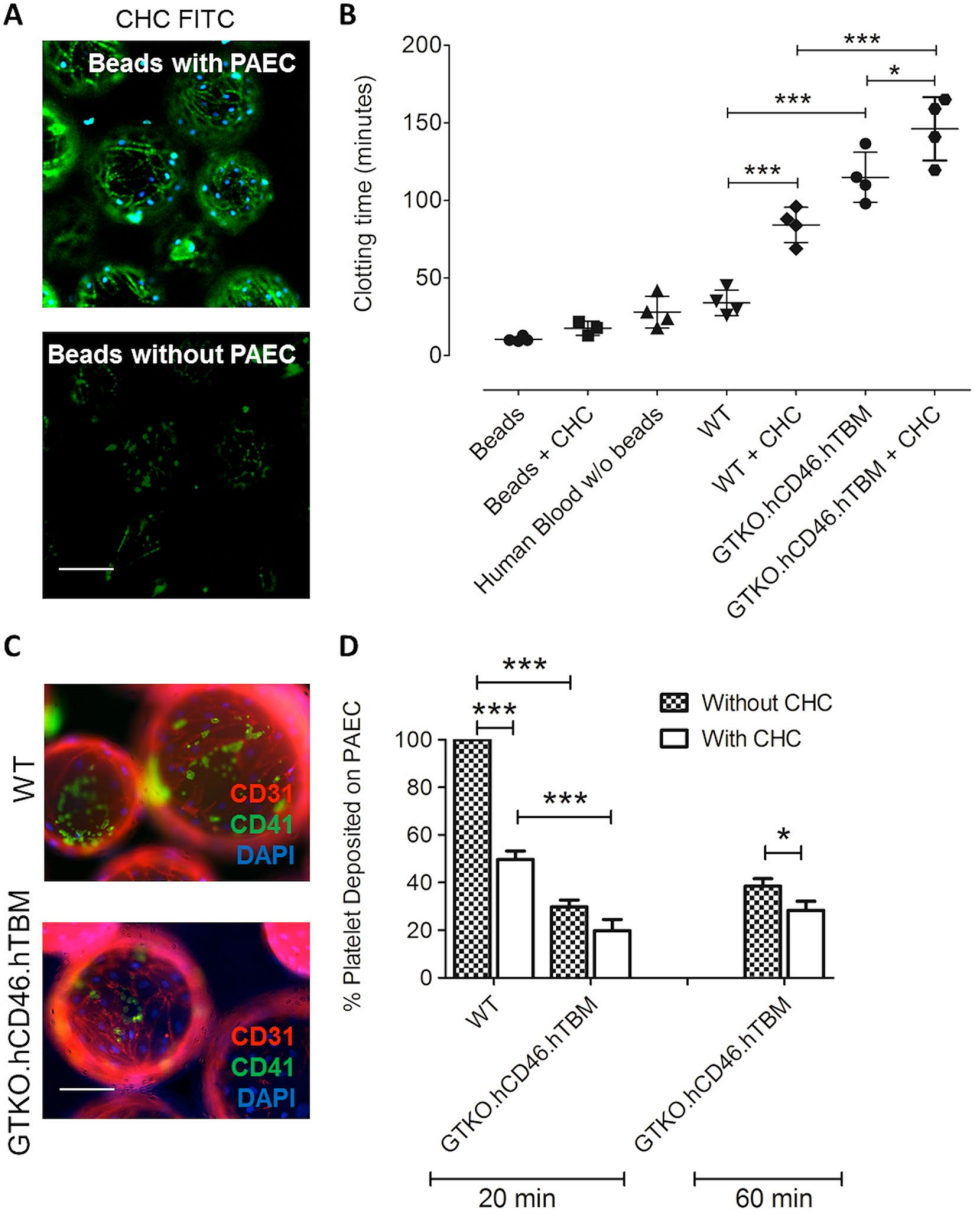
CHC coating of microbead-borne PAEC reduces clotting time of whole human blood. (**A**) Binding of FITC-labeled CHC (100 µg/ml, 15 min) to PAEC grown on microbeads (top panel), analyzed by confocal microscopy. Minimal binding of FITC-CHC to control beads without PAEC was observed (bottom panel). Nuclei were stained with DAPI. Scale bar: 100 µm. (**B**) Clotting time of non-anticoagulated whole human blood incubated with and without microbeads. Microbeads carrying GTKO.hCD46.hTBM PAEC significantly prolonged clotting time compared to microbeads carrying WT PAEC. Coating of WT or GTKO.hCD46.hTBM PAEC on microbeads with CHC significantly prolonged clotting time compared to uncoated PAEC. Statistical analysis was carried out by one-way ANOVA with Bonferroni correction (*p < 0.05, **p < 0.01, ***p < 0.001). Data are mean ± SD of four independent experiments per group. (**C,D**) CHC coating of microbead-borne PAEC reduces deposition of human platelets during incubation with human blood. (**C**) PAEC-bead samples were collected from the whole blood coagulation assays at 20 min and stained for expression of CD31 (red) and deposition of CD41-positive human platelets (green). Nuclei stained with DAPI (blue). Scale bar: 100 µm. (**D**) Platelet deposition was determined by counting the number of platelets on 20 randomly selected beads, and is presented as % platelet deposition relative to that observed with uncoated WT PAEC. Coating of WT and GTKO.hCD46.hTBM PAEC with CHC significantly reduced platelet deposition at 20 min and 60 min, respectively. GTKO.hCD46.hTBM PAEC showed significantly reduced platelet deposition compared to WT PAEC irrespective of CHC coating. Data are mean ± SD of at least three independent experiments. Significance was tested using one-way ANOVA with Bonferroni correction (*p < 0.05, **p < 0.01, ***p < 0.001).

We have previously developed an *in vitro* model in which PAEC are grown to confluence on collagen-coated microcarrier beads and mixed with freshly drawn non-anticoagulated human blood, and the time to clotting measured. This model, which mimics the interaction between the endothelial surface of a small porcine vessel and human blood^18^, was used to determine the anticoagulant effect of coating PAEC with CHC. To confirm binding of CHC, PAEC-bearing microbeads were incubated with 50–200 µg/ml FITC-labeled CHC for 15–60 min. Confocal microscopy revealed strong binding of CHC at 100 µg/ml and 15 min incubation to the cells on microbeads, whereas binding of CHC under these conditions to control beads without PAEC was minimal (Fig. 3A).

The collagen-coated microbeads without PAEC triggered rapid clotting of human blood (10.6 ± 1.6 min, Fig. 3B). Microbeads bearing WT PAEC (no CHC) prolonged clotting time to 34.0 ± 8.2 min (Fig. 3B). Microbeads with GTKO.hCD46.hTBM PAEC (no CHC) prolonged clotting time to 115.0 ± 16.1 min (p < 0.001 vs. WT (no CHC)) (Fig. 3B), consistent with previous findings^19^.

Coating of control microbeads (without PAEC) with CHC induced clotting of human blood in 17.8 ± 4.5 min (Fig. 3B). CHC coating of WT PAEC significantly prolonged clotting time (CHC 84.3 ± 11.3 min vs. no CHC 34.0 ± 8.2 min, p < 0.001) (Fig. 3B). CHC coating also further improved the hemocompatibility of GTKO.hCD46.hTBM PAEC, significantly prolonging clotting time (CHC 146.2 ± 20.4 min vs. no CHC 115.0 ± 16.1 min, p < 0.05) (Fig. 3B).

### Surface coating of PAEC with CHC inhibits binding of human platelets

The interaction between pig endothelium and human platelets leads to platelet adhesion and activation that promote clotting. Therefore, we examined the effect of CHC coating on the deposition of platelets on the surface of PAEC microbeads during incubation with human blood, before complete clot formation. The microbead-blood mixture was sampled at 20 min (WT and GTKO.hCD46.hTBM PAEC) and 60 min (GTKO.hCD46.hTBM PAEC only) and the PAEC were analyzed for CD41-positive platelet deposition by fluorescence/confocal staining (Fig. 3C). WT PAEC (no CHC) sampled at 20 min showed the highest level of platelet deposition, and were therefore selected as the comparator (100% deposition). CHC coating of WT PAEC significantly reduced platelet deposition at 20 min (49.7 ± 3.6%, p < 0.001 vs. no CHC; Fig. 3D). GTKO.hCD46.hTBM PAEC (no CHC) also showed significantly reduced platelet deposition at 20 min (29.8 ± 3.0%) compared to WT PAEC (no CHC) (p < 0.001; Fig. 3D). CHC coating of GTKO.hCD46.hTBM PAEC reduced platelet deposition at 20 min (not significant) and 60 min (CHC 28.4 ± 3.7% vs. no CHC 38.5 ± 3.2%, p < 0.001, Fig. 3D).

### Surface coating of PAEC with CHC inhibits activation of the human plasma cascade systems

**Figure 4:**
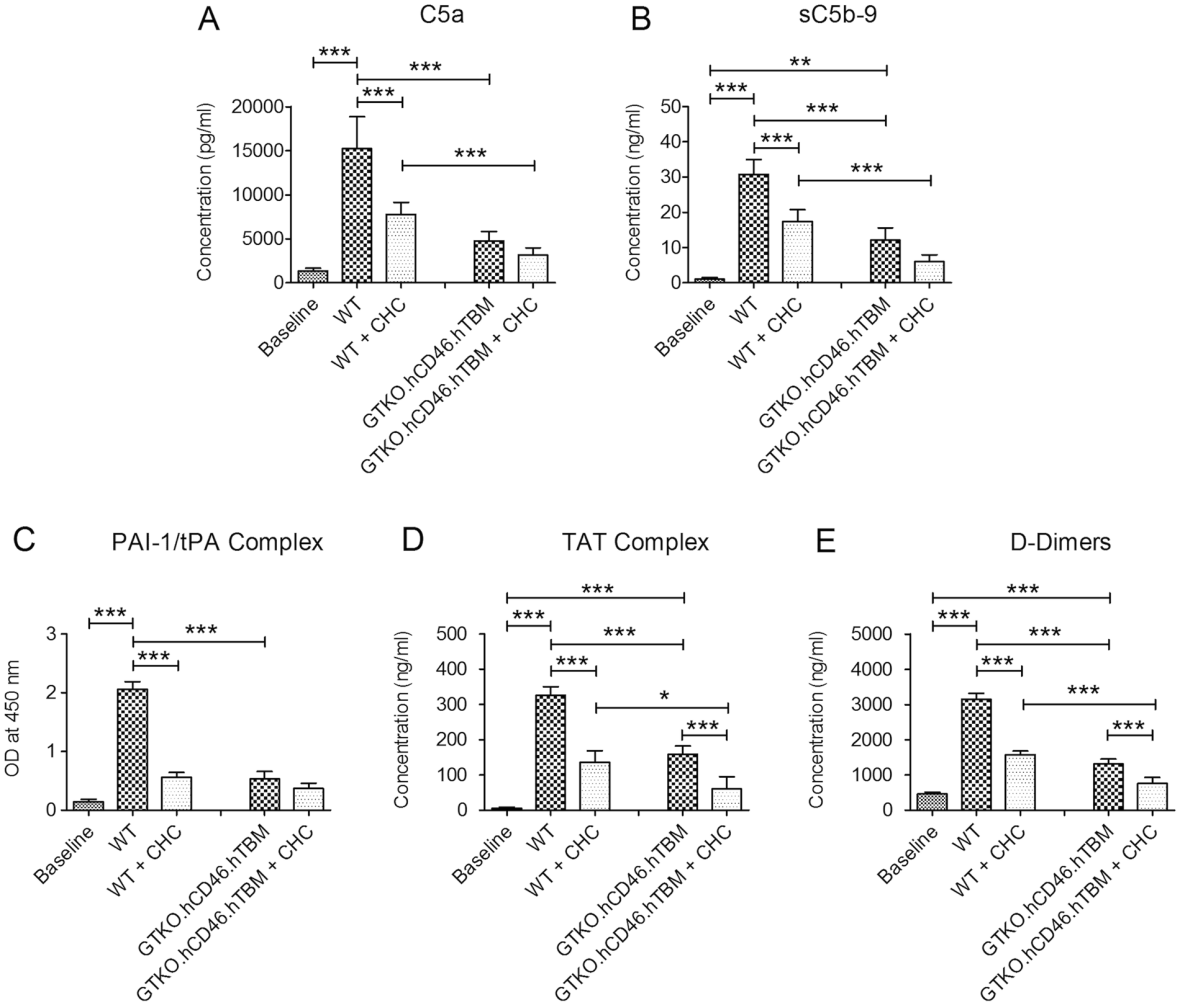
CHC coating of microbead-borne PAEC inhibits formation of soluble complement, coagulation and fibrinolytic markers during incubation with human blood. EDTA- and citrate-plasma samples were collected from the whole blood coagulation assays at 20 min and assayed by ELISA for (**A**) C5a, (**B**) sC5b-9, (**C**) PAI-1/tPA complex in EDTA-plasma and (**D**) TAT complex and (**E**) D-dimers in citrate plasma. Coating of WT PAEC with CHC significantly reduced the plasma levels of all 5 markers. GTKO.hCD46.hTBM PAEC (no CHC) induced significantly lower levels of the markers than WT PAEC (no CHC); CHC coating of the GTKO.hCD46.hTBM PAEC further reduced TAT complex and D-dimers formation. Data are mean ± SD of four independent experiments. Significance was tested using one-way ANOVA with Bonferroni correction (*p < 0.05, **p < 0.01, ***p < 0.001).

To study the effect of CHC coating of PAEC on activation of the human plasma cascade systems, EDTA- and citrate-plasma was collected from the whole blood coagulation assays at 20 min (i.e. prior to clotting) and assayed by ELISA for soluble markers of complement activation (C5a and sC5b-9), and anti-fibrinolytic activity (plasminogen activator inhibitor-1/tissue plasminogen activator (PAI-1/tPA) complexes) in EDTA-plasma and coagulation activation (thrombin-antithrombin (TAT) complexes, D-dimers) in citrate-plasma. Baseline values of all markers were measured in EDTA-plasma from blood samples taken prior to incubation with beads. All five markers were significantly increased in plasma from WT PAEC (no CHC)/human blood mixtures (C5a, sC5b-9, PAI-1/tPA, TAT, D-dimers: all p < 0.001 vs. baseline; Fig. 4A–E). CHC coating of WT PAEC significantly reduced all markers compared to plasma from WT PAEC (no CHC)/human blood (all p < 0.001; Fig. 4A–E). Plasma from GTKO.hCD46.hTBM PAEC (no CHC)/human blood mixtures showed reduced levels of all markers compared to plasma from WT PAEC (no CHC)/human blood (C5a, sC5b-9, PAI-1/tPA TAT, D-dimers: all p < 0.001) (Fig. 4A–E). CHC coating of GTKO.hCD46.hTBM PAEC reduced levels of all markers, although this was significant only for TAT complex and D-dimers (p < 0.001 vs. no CHC, Fig. 4D,E).

## Discussion

Activation of the vascular endothelium of porcine organ xenografts can be triggered by several factors, including ischemia-reperfusion, antibody binding and activation of complement. One of the most rapid changes in activated endothelium is an alteration in the configuration of the glycocalyx, with shedding of its components into the bloodstream^20, 21^. Damage to the endothelial glycocalyx causes a decrease in vascular barrier function, protein extravasation and tissue damage, and increases intravascular adhesion of leukocytes and platelets to promote blood coagulation. Here, we describe the impact of two approaches to limit the pro-inflammatory and pro-coagulant activation of porcine endothelial cells by human plasma or whole blood: pharmacological, by coating the cells with the heparin conjugate CHC; and genetic, by modifying the cells to reduce antibody binding and activation of complement and coagulation (GTKO.hCD46.hTBM).

The use of heparin as a therapeutic agent has limitations due to short half-life and weak affinity, as well as its variable anticoagulant responses. There is therefore a need of heparin conjugate preparations ensuring a prolonged half-life and increased affinity. In addition, surface immobilization of such conjugates may provide the preferred biocompatibility with heparin’s intact biological activity. Using two *in vitro* models, we showed that coating of WT PAEC with CHC markedly reduced plasma-induced cellular activation and dampened activation of the cascade systems in whole blood, resulting in a significant prolongation of clotting time. We suggest that the protective effect of CHC stems from a concentration of heparin’s anticoagulant and anti-complement activity at the endothelial cell surface. It is also conceivable that the glycocalyx was at least partially preserved due to the reduction in cellular activation, and/or protected by the CHC layer, although our data do not allow definitive conclusions on this point.

Interestingly, uncoated GTKO.hCD46.hTBM PAEC were protected from plasma and whole blood to a similar degree as CHC-coated WT PAEC. This confirms the important role of these genetic modifications in prolonging the survival of solid organ xenografts in nonhuman primate preclinical models^9^. Nevertheless GTKO.hCD46.hTBM PAEC were not completely immune to human plasma, exhibiting some loss of heparan sulfate (albeit not statistically significant) (Fig. 1A). Importantly, CHC coating on GTKO.hCD46.hTBM PAEC augmented protection against whole blood, reducing platelet deposition and thrombin generation and further prolonging clotting time. In line with previous reports^9, 22^, our results also suggest the potential benefit of heparin-based treatment following genetically modified pig organ xenotransplantation.

In summary, our data suggest that the combination of genetically modified donor pigs and treatment of xenografts with CHC may provide optimal protection against the development of a pro-inflammatory and prothrombotic milieu in the immediate post-transplant period. The unavoidable interval between organ procurement and transplantation provides an opportunity for CHC treatment; it has been shown that *ex vivo* machine perfusion of porcine kidneys with CHC results in uniform distribution of CHC binding within the vasculature^15^. A remaining question is whether the CHC coating will remain for a sufficient time post-transplant to allow recovery of the endothelium. We have addressed this question using a mouse model of syngeneic kidney transplantation in which grafts are cold-stored for 5 hrs in preservation solution prior to transplant, resulting in considerable ischemia-reperfusion injury. We found that addition of CHC to the preservation solution significantly improved post-transplant graft function at 24 hrs (Nordling *et al*., manuscript submitted). Currently, no data exist on the long-term effects of CHC treatment and its systemic effects if shed from cells in solid organ transplantation. In addition, whether the early protective effects of CHC would improve the survival of genetically modified pig xenografts remains to be determined in preclinical xenotransplantation models.

## Methods

All experiments with animals and human samples were performed in accordance with relevant guidelines and regulations. Genetically engineered pigs were generated and maintained at LMU Munich according to the German Animal Welfare Act with permission of the responsible authority (Regierung von Oberbayern; approval no. 55.2-1-54-2531-54). Human blood was drawn from healthy volunteers with informed consent and with the approval of the St Vincent’s Hospital Melbourne Human Research Ethics Committee.

### Endothelial cells

PAEC were isolated from WT or GTKO.hCD46.hTBM transgenic pig aorta harvested from euthanized pigs as described previously^19, 23^. Porcine aortic endothelial cells were used between passages 2 and 8 in all experiments.

### Immunofluorescence staining

PAEC, grown to confluence on eight-well Lab-Tek chamber slides (Milian, Switzerland), were incubated with 1:10 diluted pooled normal human plasma in 0.9% NaCl at 37 °C for 4 hrs. For CHC coating, prior to human plasma incubation, cells were treated with either starvation medium (RPMI/0.5% FBS) for 30 min at 37 °C or 100 ng/ml hTNFα (PHC3015, Biosource) for 2 hrs at 37 °C and coated with unlabeled or fluorescein isothiocyanate (FITC) labeled CHC (Corline Biomedical AB, Uppsala, Sweden) at 20–100 µg/ml in RPMI/0.5%FBS for 30 min at 37 °C. Slides were washed and fixed with 3.7% formalin for 15 min at room temperature (RT). After blocking with phosphate buffered saline (PBS)/1%BSA for 30 min at RT, slides were incubated for 60 min with mouse anti-HS FITC (10E4 epitope, H1890, US Biological Life Sciences) diluted in PBS/1% BSA. Nuclei were stained using 4′, 6′-diamidino-2-phenylindole (DAPI, Boehringer Ingelheim). After washing, the slides were mounted with glycergel (C0563; Dako). The stained slides were then examined using a Nikon A1R confocal microscope. Mean fluorescence intensity (MFI) was calculated using Image J software.

### Cell ELISA

A modified whole-cell ELISA was used to measure xenogeneic complement-mediated responses, similar to methods described previously^24^. PAEC were grown to confluence in 96-well plates and washed twice with PBS/0.05% Tween20. Cells were treated with either starvation medium for 30 min at 37 °C or hTNFα (100 ng/ml) for 2 hrs at 37 °C, and incubated with CHC at 100 µg/ml diluted in RPMI/0.05%FBS for 30 min at 37 °C followed by washing. Pooled human plasma diluted in 0.9% NaCl (1:10) was added to the cells and incubated at 37 °C for 4 hrs. After washing, cells were fixed in 3.7% formalin for 15 min at RT, washed, and blocked with PBS/1% BSA for 60 min at RT. Rabbit anti-human C3b/c FITC (A0062; Dako), mouse anti-human C5b-9 FITC (clone aE11; HM2167F, Hycult Biotech), and mouse anti-human CD62E FITC (clone 1.2B6, MCA883F, AbD Serotec) were diluted in PBS/1% BSA and incubated for 60 min at RT followed by three washes. Subsequently, HRP-conjugated rabbit anti-FITC (P0404; Dako) diluted 1:1000 in PBS/1% BSA was incubated for 30 min at RT followed by TMB (3,3′,5,5′-Tetramethylbenzidine) for color development and stopped by 2N H_2_SO_4_. Color development was quantified at 450/540 nm using a FLUOstar Omega microplate reader.

After plasma treatment, supernatants from the assay were collected in sodium citrate (3.2%) tubes and assayed for D-dimer levels using a commercial ELISA kit (ASSERACHROM® D-DI, REF 00947, Diagnostica Stago, Asnieres) according to the manufacturer’s protocol.

### Endothelial cell culture on microcarrier beads

PAEC were cultured on collagen-coated Biosilon microcarrier beads as described previously^18, 19, 25^. Briefly, Biosilon microcarrier beads (polystyrene, 160–300 μm) were coated with 100 μg/ml bovine collagen I (sc-29009, Santa Cruz Biotechnology) in 0.2% acetic acid for 60 min at RT. PAEC harvested from one 175-cm^2^ flask (1.5–1.7 × 10^7^ cells) were mixed with 5–7 ml collagen I-coated beads in 100 ml medium M199 (Sigma-Aldrich) supplemented with 10% heat-inactivated FBS (Thermo Fisher Scientific), 1% penicillin/streptomycin (Thermo Fisher Scientific), 1% L-glutamine (Life Technologies), 1.2% supplement mix-II (C-39216, PromoCell), and 5000 U/ml heparin (Heparinum natricum, Liquemin®), in a 500 ml stirred culture flask. The beads were stirred for 5 min at 50 rpm with intervals of 45 min using a Cellspin control unit (Integra Biosciences) at 37 °C. After overnight incubation, 75 ml of medium M199 plus supplements was added to the flask and incubated for another 24 h. Then, the volume of the medium was adjusted to 320 ml with RPMI (Sigma-Aldrich) plus supplements. Every 48 h, 100 ml of the culture medium was exchanged with RPMI plus supplements. Cell confluence after 5–7 days was estimated by analysis of DAPI staining using a Nikon A1R confocal microscope.

### *In vitro* human blood clotting assay

2 ml beads with confluent cells in a 10 ml polypropylene tube were washed with RPMI medium without supplements to remove heparin, and then incubated with starvation medium for 30 min at 37 °C. Subsequently, the PAEC/beads were either untreated or coated with FITC-labeled or unlabeled CHC in RPMI-0.5% FBS for 15 min at 37 °C. After coating, the beads were washed and incubated with freshly drawn, non-anticoagulated whole human blood at a bead-to-blood volume ratio of 1:4 (i.e. 2 ml beads and 8 ml whole blood) that closely mimics the *in vivo* small vessel endothelial surface-to-blood volume ratio. Human blood was drawn from healthy volunteers (two individual donors [blood groups: A-positive and O-positive] were used; single donor per experiment). The bead-blood mixture tubes were incubated at 37 °C with gentle rocking, and monitored for clotting time without any interruption. For sampling, experiment tubes were interrupted at designated time intervals (i.e. before the onset of clotting, after 20 min for WT PAEC ± CHC; after 20 min and 60 min for GTKO.hCD46.hTBM PAEC ± CHC) and 2 ml samples were withdrawn to retrieve PAEC-covered beads and EDTA-plasma.

Sampled beads were washed 3 times with PBS containing MgCl_2_ and CaCl_2_ and fixed for 20 min in 3.7% formalin. Following washing, the beads were stained with rat anti-porcine CD31 (MAB88371, R&D Systems) plus goat anti-rat IgG Alexa Fluor 546 (Thermo Fisher Scientific) and mouse anti-human CD41 FITC antibody. The coverage of beads with PAEC, and CD41-positive platelet deposition on PAEC, were determined by counting the number of cell nuclei (DAPI) and CD41-positive platelets on 20 randomly selected beads, on a defined rounded bead surface using a Nikon A1R confocal microscope.

### ELISAs for measurement of complement and coagulation markers in plasma

Using ELISA, complement activation (C5a, soluble (s)C5b-9), and anti-fibrinolytic activity (PAI-1/tPA complexes) markers in EDTA-plasma and coagulation activation markers (TAT complexes, D-dimers) in citrate-plasma were measured. C5a, TAT complexes and D-dimers were measured using commercial ELISA kits (C5a: human C5a Duoset, DY2937, R&D Systems; and TAT complex: Enzygnost® TAT micro, OWMG15, Dade Behring; D-dimers: Diagnostica Stago), according to the manufacturer’s protocol. PAI-1/tPA complex was measured using mouse antihuman PAI-1 (HM2181, Hycult Biotech) and biotinylated rabbit antihuman tPA (ASHTPA-GF-BIO, Molecular Innovations) antibodies. sC5b-9 in plasma was detected using an in-house developed ELISA with mouse anti-human C5b-9 (Hycult Biotech) and biotinylated mouse anti-human C6 (A706, Quidel) antibodies.

### Statistical Analysis

Data are shown as mean ± standard deviation. They were analyzed using GraphPad Prism 5.0 (GraphPad Software). Significance was tested using unpaired Student’s t test (2-tailed) or one-way analysis of variance with Bonferroni correction (*P < 0.05, **P < 0.01, ***P < 0.001).
